# Rapidly Learning Generalizable and Robot-Agnostic Tool-Use Skills for a Wide Range of Tasks

**DOI:** 10.3389/frobt.2021.726463

**Published:** 2021-12-14

**Authors:** Meiying Qin, Jake Brawer, Brian Scassellati

**Affiliations:** Yale Social Robotics Lab, Department of Computer Science, Yale University, New Haven, CT, United States

**Keywords:** robot tool use, tool manipulation, tool improvisation, tool substitution, platform-agnostic representations, learning from demonstration, generalizability

## Abstract

Many real-world applications require robots to use tools. However, robots lack the skills necessary to learn and perform many essential tool-use tasks. To this end, we present the TRansferrIng Skilled Tool Use Acquired Rapidly (TRI-STAR) framework for task-general robot tool use. TRI-STAR has three primary components: 1) the ability to learn and apply tool-use skills to a wide variety of tasks from a minimal number of training demonstrations, 2) the ability to generalize learned skills to other tools and manipulated objects, and 3) the ability to transfer learned skills to other robots. These capabilities are enabled by TRI-STAR’s task-oriented approach, which identifies and leverages structural task knowledge through the use of our goal-based task taxonomy. We demonstrate this framework with seven tasks that impose distinct requirements on the usages of the tools, six of which were each performed on three physical robots with varying kinematic configurations. Our results demonstrate that TRI-STAR can learn effective tool-use skills from only 20 training demonstrations. In addition, our framework generalizes tool-use skills to morphologically distinct objects and transfers them to new platforms, with minor performance degradation.

## 1 Introduction

Imagine a robot designed to perform household chores. Such a robot will encounter many tasks requiring the use of a wide variety of tools, for example, cutting and stirring ingredients to help with cooking, scooping pet food to care for family pets, and driving screws and hammering nails to assist with house maintenance. In order to be a help and not a hindrance, such a robot would need to be capable of rapidly learning a wide assortment of tasks. In addition, given the complexity of household chores and the diverse range of objects that could be encountered, a robot should be able to generalize learned skills to novel tools and manipulated objects without needing to be retrained. Finally, one might wish to leverage learned skills from other users or transfer a library of accrued skills to a new robot without retraining.

A framework that enables such capabilities would have applications that extend far beyond the household. The search-and-rescue and disaster cleanup domains, for example, could benefit from such capabilities. Since these scenarios can be highly unpredictable and resource-limited, the robot should be able to both learn the appropriate tool-use skills rapidly and substitute learned tools for improvised alternatives. In addition, the ability to transfer learned skills to other robot platforms will enable rapid deployment of new models to assist or to replace a damaged teammate, regardless of different robot kinematic configurations.

This study focuses on learning and applying tool-use skills in a *task-general* manner (i.e., to handle a wide variety of tasks without predefined information for each specific task). In the course of a *task*, a single action is taken with objects in order to achieve a particular goal. The *objects* include a *tool*, an object that is “graspable, portable, manipulable, and usually rigid” (Gibson, 1979), and a *manipulandum*, an un-grasped object being manipulated by the tool. Similar to previous tool-use studies, we only consider tool-use tasks involving the following: 1) tools and manipulanda that are unjointed rigid bodies, 2) the use of contact forces to deterministically change the state of the manipulandum, and 3) a goal that can be accomplished with a single tool action, rather than a series of actions.

We report on a task-general integrative tool-use framework called TRansferrIng Skilled Tool Use Acquired Rapidly (TRI-STAR). The framework includes components such as perception, 3D mesh scanning, tool-use skill learning, and tool-use skill generalization. These components collectively endow a robot with three capabilities, or *Stars*, aimed at solving challenging and commonplace problems in robot tool use. Star 1 is the ability to learn and apply a wide range of tasks with minimal training. Star 2 is the ability to generalize the tool-use skills learned with trained tools (i.e., *source*, color-coded green in the figures and movies) in Star 1 to both novel (i.e., *substitute*, color-coded blue) tools and manipulanda with no additional training, which is *object substitution*. Star 3 is the ability to transfer learned skills directly to other robot platforms (color-coded yellow), which is *platform generalization*.

### 1.1 Task-Oriented Approach to Tool-Use Skills

Tool-use skills are actions composed of two components: motor skills and contact poses. Motor skills concern the tool trajectory (i.e., a time series of poses of a tool) and dynamics (i.e., the forces required for successful tool use). The contact poses consider tool–manipulandum contact poses and gripper–tool contact poses or graspings, which are dependent on the tool–manipulandum contact poses. While previous studies generally focus on one aspect of the skills, our system considers multiple skills, or the minimum set of tool-use skills that enables a robot to use a tool, which includes the tool trajectory and tool–manipulanda contact poses (henceforth referred to as contact poses).

While some tool-use studies are *tool-oriented* in that they seek to model tool use for a specific tool or class of tools (e.g., Stoytchev, 2005; Sinapov and Stoytchev, 2008; Jamone et al., 2016; Zech et al., 2017), we opted for a *task-oriented* approach (Detry et al., 2017; Kokic et al., 2017) that learns associations between tasks and tool-use skills. This is a more natural framing of the problem as tool use is not driven by the tool itself but instead by the task. To illustrate, the actions taken using a spoon on a piece of cake differ when one cuts the cake into smaller pieces or scoops a piece in order to eat it. In both tasks, the tool (the spoon) and even the manipulandum (the cake) are the same, so differences in how the tool is used can only be explained by the differences in the tasks. In a tool-oriented approach, the tool would have uniquely determined a single action for both steps.

**FIGURE 1:**
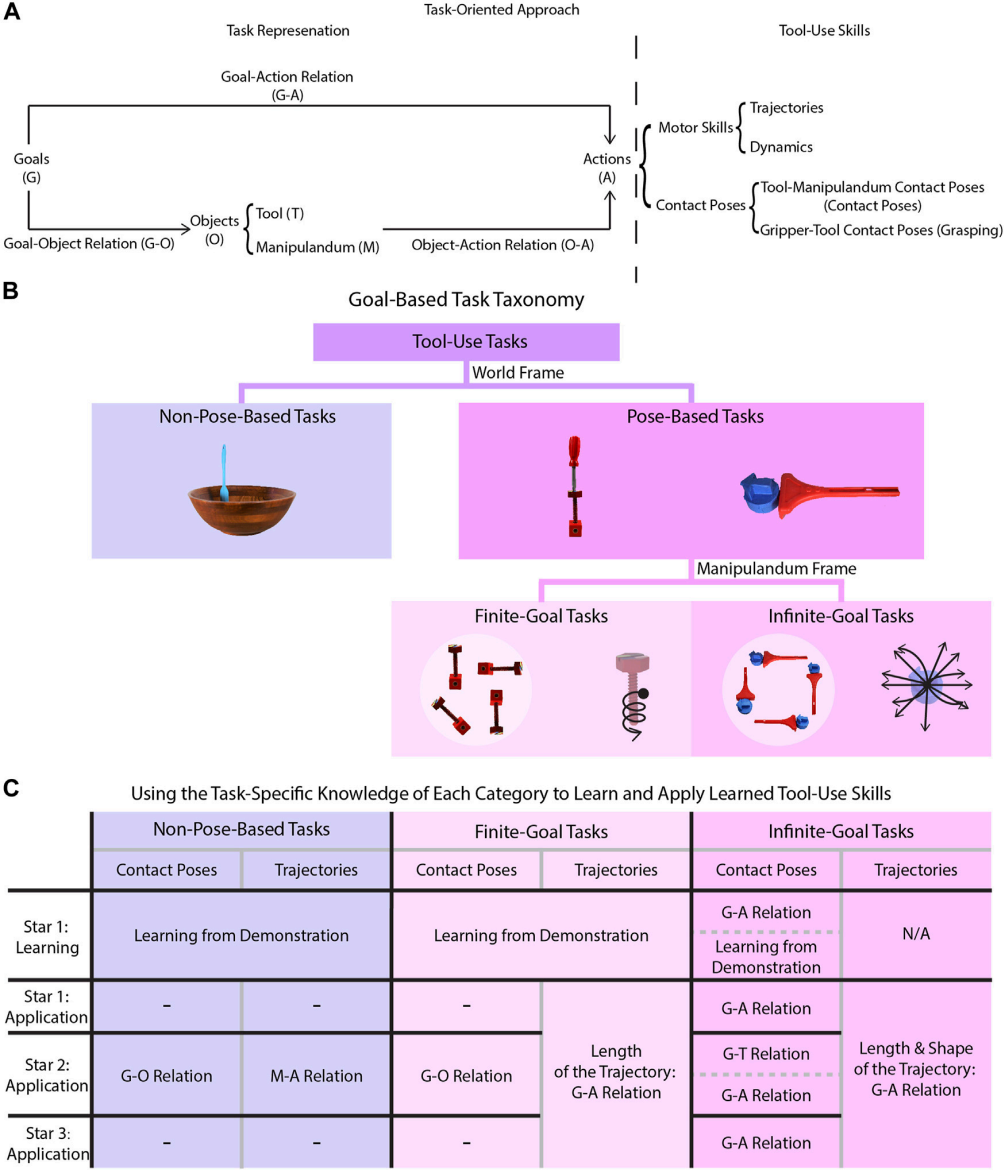
Algorithm overview. **(A)** is a diagram depicting the task-oriented approach to tool-use skill learning and application. The causal relations between the goals, objects, and actions are represented by the directed edges of the diagram. **(B)** depicts the task taxonomy whose structure emerges when observing goal-based motion primitives from different frames of reference. **(C)** is a chart summarizing taxonomic knowledge for each combination of task category and Star during the tool-use skill learning or application process. Each cell specifies the specific task-specific knowledge relation needed, if any, denoted by the abbreviations G, O, A, T, and M defined in **(A)**.

In a task-oriented approach, goals, objects, and actions are connected through specific relationships. By our definition of tasks, the relationship between these three components is inherently causal with goals as the primary causal antecedent (as depicted in Figure 1A); a goal causes an agent to select features of objects (e.g., the goal of cutting requires a tool to be sharp), and the objects and the goal determine a precise action to be taken (e.g., the desired position of a block determines how it should be pushed, and the size of a bowl influences the radius of a stirring motion). While these goal–object relations, goal–action relations, and object–action relations, respectively, may differ across tasks, they remain constant across instances of a particular task and are useful when learning and generalizing tool-use skills.

Specifying these three relations for each task is impractical and learning these relations for each task can be data intensive. However, the causal structure of this approach implies that tasks with similar goals also share common features of each type of relation. Therefore, we compiled a task taxonomy (see Section 2.1.1) that categorizes tasks based on their goals with respect to manipulanda as shown in Figure 1B and summarized the common features of each relation in each category as shown in Figure 1C, which we called *taxonomic knowledge*. The advantage of utilizing taxonomic knowledge is that specific information does not need to be manually specified for new tasks when either learning a task or applying the learned tool-use skills. In this way, taxonomic knowledge can help to reduce the training data needed.

### 1.2 Star 1: Learning and Applying Task-General Tool-Use Skills

Star 1 is the ability to learn tool-use skills and apply them to complete the same task with new configurations using a source tool and manipulandum. In this section, we first describe relevant studies, though they often ignored contact poses entirely or utilized a simplistic contact pose representation or did not utilize the goal–action relations to apply skills. We then describe the challenges in learning tool-use skills and briefly describe the tests we conducted.

Studies focusing on motor skills ignored the learning of contact poses, though they were applied to tool-use tasks such as swinging tennis rackets (Ijspeert et al., 2002), batting (Peters and Schaal, 2006), playing ball-in-a-cup (Kober et al., 2008) or table tennis (Muelling et al., 2010), pouring (Pastor et al., 2009; Rozo et al., 2013), writing letters (Lioutikov et al., 2017) or digits (Droniou et al., 2014), and peg-hole insertion (Gao and Tedrake, 2021) with methods such as dynamical movement primitives (Schaal, 2006; Ijspeert et al., 2013) or probabilistic movement primitives (Paraschos et al., 2013). For example, in the peg-hole insertion study, experimenters hard-coded the contact poses so that the end of a peg should align with the top of a hole vertically when learning the peg-hole insertion task.

Studies that did not ignore contact poses (Kemp and Edsinger, 2006; Hoffmann et al., 2014) utilized the tool tip as a simplified representation of the contact area. Yet, in practice, the contact area can comprise any arbitrary area at any location on a tool, such as the tip of a screwdriver, the blade of a knife, the face of a hammer, or the concave surface of a ladle. Moreover, with such a simplification, the relation between the tool and the manipulandum is reduced to be the angle of contact, which is insufficient for tasks like screw-driving; a screwdriver should contact a screw not only perpendicular to the head of the screw but also with the correct rotation about the tip axis. Additionally, such simplified representations cannot account for tasks that may have multiple viable contact poses; a hammer may approach a nail from infinitely many orientations about the head axis of the nail and thus have an infinite number of viable contact poses.

While the aforementioned studies did not incorporate goal–action relations into the action generation process, studies that focused on these relations did not consider action generation. Two previous studies (Sinapov and Stoytchev, 2007; Stoytchev, 2008) learned how predefined linear end-effector trajectories of different tools lead to positional changes of a manipulandum. Another study (Zech et al., 2017) attempted to learn relationships between goals and contact poses to aid in tool selection but predefined a contact pose template. Other studies (Moldovan et al., 2013; Gonçalves et al., 2014A; Gonçalves et al., 2014B; Dehban et al., 2016) learned these relations from a probabilistic approach but also with predefined end-effector trajectories.

Star 1 learns and applies tool-use skills by locating the task in the taxonomy and utilizing taxonomic knowledge (i.e., the goal–action relations) identified by its category. We demonstrated seven tasks (knocking, stirring, pushing, scooping, cutting, writing, and screw-driving) that learned with a small number of training samples and tested different types of tool-use skills. This range of tasks tested the learning and application of tool-use skills given different task types, such as stirring, screw-driving, and pushing, each corresponding to a type defined in the taxonomy we describe in detail in the methodology.

### 1.3 Star 2: Task-General Object Substitution

Star 2 is the ability to generalize learned tool-use skills from source to substitute tools or manipulanda that can complete the task, including objects that share a common geometric template (geometrically similar objects, e.g., mugs differing in shape and size as in the study by Brandi et al. (2014)) or share no common form-factor (geometrically distinct objects, e.g., pushing an object with a cake-cutter rather than a toy rake). To generate actions, an object-substitution algorithm must adjust learned trajectories for tasks such as stirring in a smaller container and produce contact poses. The contact poses for many tasks can be obtained by finding the mapping between the source and substitute objects based on features for tasks such as cutting, but for some tasks like pushing, the contact poses are determined by goals of the tasks. Similar to previous tool-use studies, we focused on geometric features only.

Many previous studies employed task-specific approaches that limited the robot’s ability to improvise tools using objects that share common form-factors. Some of these approaches required hand-engineered information to find a mapping for each task (e.g., Hillenbrand and Roa, 2012; Brandi et al., 2014; Stückler and Behnke, 2014). Providing hand-engineered information for each task exhibits two disadvantages. First, algorithms requiring hand-engineered information constrain their user-friendliness for naïve end-users who lack the knowledge to train these algorithms adequately. Second, engineering information for each task is time-consuming and impractical in real-world settings requiring the use of many tools.

Other approaches that can accommodate tools of various shapes usually require prohibitively large amounts of data per task. For example, over 20,000 training examples were needed to learn and generalize in the pushing task (Xie et al., 2019); 18,000 simulated tools were used to generalize tool use in a sweeping and nail-hammering task (Fang et al., 2020); 5,000 vectorized representation tools were used to train a neural network to generalize tool-use in the scraping, cutting, and scooping tasks (Abelha and Guerin, 2017; Gajewski et al., 2019). Acquiring a large training sample set is infeasible when tasks need to be learned rapidly or when many tasks need to be learned. Moreover, these studies only considered tool substitutions but not manipulandum substitutions, limiting their applicability to many real-life tool-use applications.

Star 2 performs object substitution by adjusting tool-use skills learned by Star 1, using all three relations comprising taxonomic knowledge without additional training. While the goal–action relations assisted the generation of actions to different task configurations in the same way as in Star 1, the two object-related relations help to generate contact poses and adjust learned trajectories. This ability to adapt trajectories to accommodate substitute objects and the ability to perform tool and manipulandum substitution are two advantages of our approach that are not typically considered in other studies. We evaluated Star 2 with five tasks (knocking, stirring, pushing, scooping, and cutting). The substitute objects differed from the source objects in size, shape, or a combination of both. We also tested trajectories requiring adjustments based on geometric features of the manipulanda (e.g., stirring and cutting), goals (e.g., pushing), and trajectories requiring no adjustments (e.g., hammering).

### 1.4 Star 3: Transferring Tool-Use Skills to Other Robot Platforms

Star 3 is the ability to transfer tool-use skills to other robot platforms. This requires a robot-independent representation of tool-use skills. Although learning trajectories and dynamics in the joint state space is common in learning motor skills, such representation makes it challenging to transfer learned skills to robots with different hardware configurations. Learning in the Cartesian space is more conducive to cross-platform transfer, though it suffers from practical limitations.

When learning in the Cartesian space, prior tool-use studies (e.g., Fitzgerald et al., 2019; Xie et al., 2019) used the gripper pose as a proxy for the tool pose to simplify the perception problem. In these studies, rather than learning tool–manipulandum contact poses and tool trajectories, the gripper–manipulandum relative pose and gripper trajectories were used to learn tool-use skills. Using gripper poses assumes that the grasps of a tool remain consistent across training and testing regimes, which is difficult to ensure outside of a controlled lab setting even on the same model of robot. When such an assumption cannot be met and a robot needs to grasp a tool differently, workarounds are sometimes employed, such as treating learned tools as novel (Sinapov and Stoytchev, 2008; Mar et al., 2017), which complicates the skill transfer process.

In contrast, tool poses are a flexible and direct representation for tool-use skills. Such a representation is not tied to any particular robot configuration and does not require grasping consistency within or across platforms. This flexibility enables a robot to perform tool-use skills with different grasps of the same tool. Crucially, this flexibility also extends to transferring skills to other robot platforms.

Star 3 performs tool-use skill transfer from a source robot to a substitute robot by leveraging our platform-agnostic representation of tool-use skills. The strength of using such a representation is that it updates a common representational schema (i.e., Cartesian end-effector trajectories) in a simple way but nevertheless greatly impacts the flexibility and generalizability of tool skills. The process of applying the skills is otherwise the same as in Star 1. We tested the transfer of tool-use skills learned using a Universal Robotics UR5e arm to both a Baxter robot and a Kuka youBot robot with six tasks (knocking, stirring, pushing, scooping, cutting, and writing). These three robots have different degrees of freedom (DoF) and are kinematically distinct. UR5e has 6 DoF, and one arm of Baxter has 7 DoF, which allows the robot to pose its end-effector freely in the 3D space. YouBot without the mobile base has only 5 DoF, which thus limits the robot’s ability to reach arbitrary poses. Depending on conditions, a robot might abort execution or slightly adjust a trajectory if it cannot be fully executed.

## 2 Materials and Methods

The TRI-STAR framework focuses on learning geometrically based tool-use skills *via* learning from demonstration with position control^1^. We first introduce and summarize the representational schemas we use throughout the system, which include the goal-based task taxonomy, trajectory, and contact pose–based tool skills, and our 3D model and 6D pose-based object representation. Subsequently, we detail the three Stars enabling the primary capabilities of our system.

### 2.1 Representations

#### 2.1.1 Task Representation: Goal-Based Task Taxonomy

We developed a taxonomy of tasks to assist learning and application of tool-use skills. Our goal-based taxonomy (Figure 1B) recognizes two fundamental task types using goals referenced in the world frame. In our study, we focus on tasks with goals described by pose changes of the manipulandum as they can be easily perceived *via* the depth cameras. Since the goals for everyday tool-use tasks generally require simple motions of the manipulanda, one screw axis can be used to characterize the shape of a goal-directed motion primitive. Non-Pose-Based Tasks are tasks with zero screw axes which represents the case where the pose of a manipulandum (e.g., a bowl) is not changed as a result of the tool usage (e.g., stirring liquid in the bowl) in the world frame. Pose-Based Tasks are tasks with non-zero screw axes such that the pose of a manipulandum changes as a result of tool usage, though two further subdivisions emerge when observing in the manipulandum frame. Finite-Goal Tasks such as screw-driving are tasks where a unique screw axis in the manipulandum frame exists to describe goal–action relations while there are still infinitely many goals in the world frame. Infinite-Goal Tasks, in contrast, like pushing a toy with a rake to the desired location, have infinitely many screw axes in the manipulandum frame to represent goals.

Taxonomic knowledge summarizes the characteristics of each task type which includes goal–action relations, goal–object relations (including goal–tool relations and goal–manipulandum relations), and object–action relations (including tool–action relations and manipulandum–action relations). As shown in Figure 1C, learning the tool-use skills in Star 1 is consistent in all three types of tasks, except that Infinite-Goal Tasks require modifications based on the goal–action relations. The contact pose required when completing a pushing task, for example, depends heavily on the goal pose of the manipulandum. Applying tool-use skills, in contrast, requires different task-specific information depending on task type and the Star, which are summarized in Figure 1C. The goal–action relations specify how actions should be updated based on the goal, which are crucial for Pose-Based Tasks because they determine, for example, the length of the trajectory when driving a screw into a thick piece of wood versus a thinner piece. The goal–object relations specify which object features are relevant for achieving the goal, such as a sharp edge of a tool in the case of a cutting task. These relations are important for generating contact poses for object substitution for all tasks except the Infinite-Goal Tasks. These tasks do not require goal–manipulandum relations but require goal–action relations since the contact pose depends on, for example, where the manipulanda should be pushed to in a pushing task. The object–action relations dictate how the actions should be updated based on different object features, which are relevant for the Non-Pose-Based Tasks such as stirring where the radius of a stirring trajectory is dependent on the size of the container containing the mixture being stirred. These relations capture common features across tasks within each category of the taxonomy and can be used to guide the learning, application, and transferring of tool-use skills to substitute objects.

#### 2.1.2 Tool-Use Skill Representation: Trajectory and Contact Poses

**FIGURE 2:**
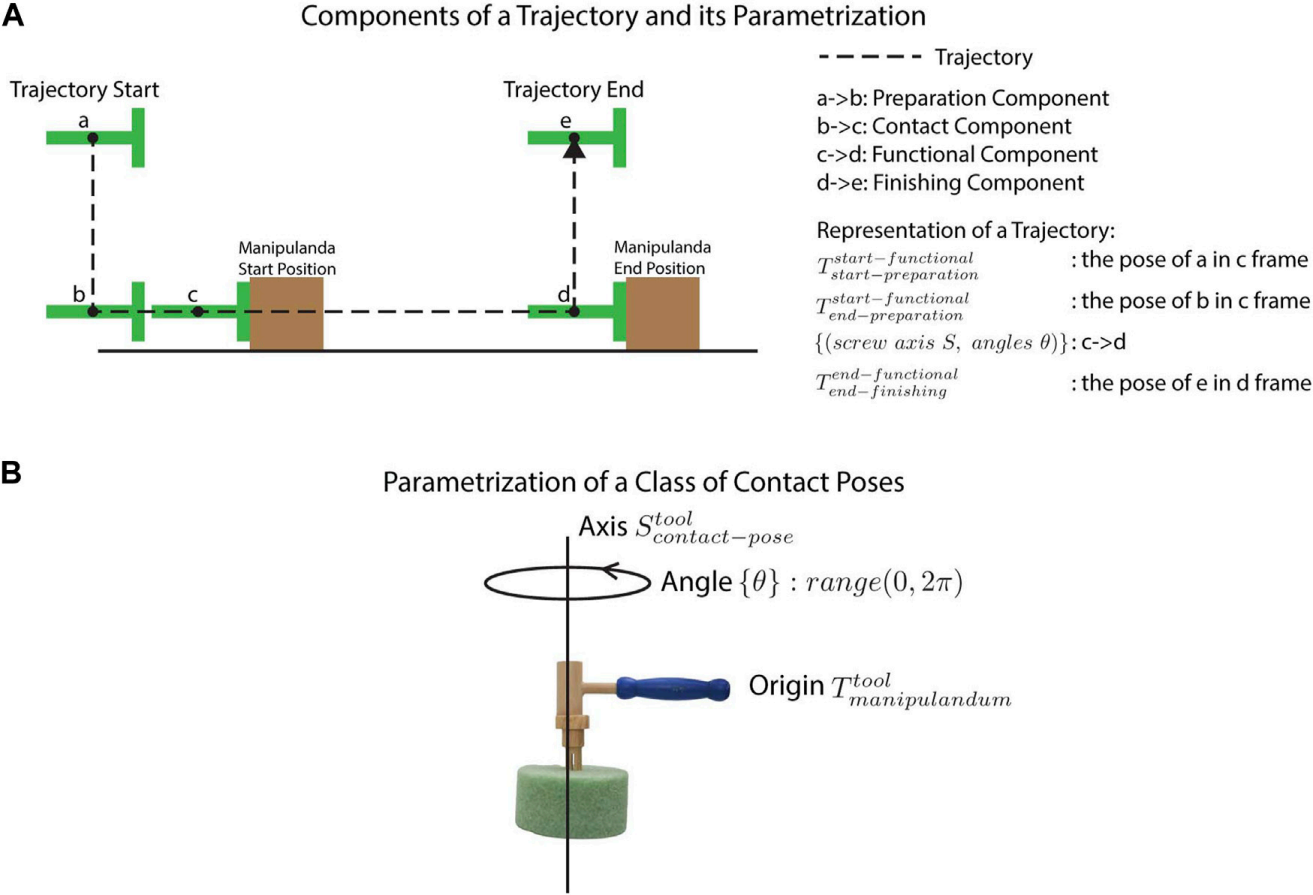
Star 1 illustrations. **(A)** depicts the four component trajectories that comprise a hypothetical demonstration of a pushing task. **(B)** depicts the parametrization of a contact pose using a nail-hammering task as an example.

A trajectory consists of four components as shown in Figure 2A: 1) the preparation component, which brings the tool in close proximity to the manipulandum, 2) the contact component, which initiates contact with the manipulandum, 3) the functional component, which acts on the manipulandum, and 4) the finishing component, which moves the tool away from the manipulandum, terminating the trajectory. The main part of the trajectory is the functional component. We represent this component using screw axis representations which are compact and easily adapted for tool use. Although we also included other components, we consider such components peripheral to the tool skill proper, and thus, they are not the focus of this study. Keeping with other tool-use studies that either completely ignore such components or hard-code them (e.g., Sukhoy et al., 2012), we represented these components simply using trajectory end points.

We represent the functional components with a series of segments {(screw axis S,  angles θ)} with each segment parametrized with exponential representations of a pose change. The advantage of such representation is twofold. First, since the screw axis includes all six DoF, no coupling between dimensions is needed as in previous methods (Schaal, 2006; Ijspeert et al., 2013; Paraschos et al., 2013). Second, in accordance with other representation schemes, trajectories can also be easily rescaled and rotated. Such representation may not be ideal for other robot manipulation tasks such as pick and place, where learned trajectories are flexibly warped based on different start and goal poses. However, this representation is suitable for the tool-use domain, where trajectories may need to be warped in a structured way based on taxonomic knowledge (e.g., to adapt a learned straight trajectory to push along a curved one required by the goal) or extended along the shape outlined by the screw axis, such as when driving the same screw into boards of different thicknesses.

The contact poses are represented with equivalence classes of poses, $\left\{T_{man}^{tool}\right\}$ , that treat all poses formed from rotating around some axis as being equivalent. This is a uniform representation for finite contact poses such as driving screws and infinite contact poses such as nail-hammering. Each element $T_{man}^{tool}$ is a manipulandum pose in the tool frame (i.e., the tool frame is the pose of the tool when initiating contact with the manipulanda). Such representation is able to accommodate contact areas of any shape located anywhere on a tool and a manipulandum and represent any orientation between the two objects. The transformations in the same class can be obtained by rotating about an axis $S_{cp}^{tool}$ . As a result, a class of contact poses (shown in Figure 2B) is parameterized as an axis $S_{cp}^{tool}$ , a transformation $T_{man}^{tool}$ as the origin, and a group of angles {θ} such that a viable contact pose can be obtained by rotating an angle θ about the axis $S_{cp}^{tool}$ starting from $T_{man}^{tool}$ . In this way, this class can represent a unique contact pose (i.e., a unique angle which is zero), limited contact poses (i.e., a limited number of angles), or an infinite number of contact poses (i.e., the angles within a range).

#### 2.1.3 Object Representation: 3D Models and 6D Poses

TRI-STAR is designed for a robot to be able to utilize novel tools without prior training. In order to accomplish this, the algorithm requires the robot to obtain 3D models of the novel objects under consideration. We used Microsoft Azure RGB-D cameras, which are commonly used and relatively inexpensive sensors, to obtain raw partial 3D point clouds. With the relatively low fidelity of perceived partial point clouds, available methods could not obtain full 3D models of sufficiently good quality. Therefore, it was necessary to design a pipeline to fit our needs.

This pipeline begins with first mounting an object in the robot’s end-effector. The robot can then rotate its end-effector around an arbitrary axis to ensure that both the back and the front of the object are visible to the 3D camera. A series of raw point clouds are obtained while the robot steps through the trajectory. The background in the point clouds is then pruned to obtain the partial point clouds of the objects. Given the pose of the end-effector at each step, the partial point clouds are merged by transforming these point clouds to the initial pose. To account for noise, we optimize the rotation axis represented as a screw axis S using the Han–Powell quasi-Newton method by minimizing the sum of the Euclidean distances between the bounding boxes of the partial point clouds and the bounding box of the merged point cloud. As parts of the objects are occluded by the robot’s own gripper, the robot obtains two such merged scans and registers them to create the final complete scan. Supplemental scans using Autodesk Recap^2^ photogrammetry software were also used to obtain point clouds for objects that are challenging for the robot to grasp. Although we attempted to design the entire process to be autonomous, the grasping during scanning and tool use requires an experimenter to assist with mounting an object to the gripper.

To obtain smoothed triangle meshes, the models are post-processed automatically with a script using meshlabxml^3^, a python interface to MeshLab^4^, similar to a previous study (Gajewski et al., 2019). The point clouds are upsampled with Poisson-disk sampling with input 5,000, meshed with Ball-Pivoting, smoothed with Taubin smoothing, and the holes filled with the default settings. The meshes are then centralized and realigned based on their minimum bound boxes.

We used a non–marker-based perception system and estimated the pose of the objects from raw sensor input. Two Azure devices are placed on the two sides of the workspace to capture a complete point cloud representation of the workspace. Background and foreground point clouds are retrieved from both sensors. The workspace is isolated, and the desktop is removed with random sample consensus (RANSAC; Fischler and Bolles, 1981) from these point clouds. To obtain a partial point cloud of the manipulanda, the background is subtracted from the foreground point clouds. The pose of the object in the world frame $T_{manipulandum}^{world}$ is obtained by rigid registration between the partial point cloud and the full 3D model. The pose with a higher fitting score, measured by calculating the ratio of inlier point correspondences over the total number of target points, is chosen. If the scores from both sensors are similar, the averaged pose is used.

The method of obtaining tool poses in the end-effector frame is similar to the method above, except for the extra step of removing points belonging to the gripper to isolate the tool. The pose of the tool in the end-effector frame is then obtained with $T_{tool}^{ee}={\left(T_{ee}^{world}\right)}^{-1}\times T_{tool}^{world}$ where × is matrix multiplication, and the superscript represents matrix inversion, given the perceived pose of the end-effector in the world frame $T_{ee}^{world}$ and the perceived tool pose $T_{tool}^{world}$ . Similar to previous tool-use studies, we assume a fixed grasp for a tool once it is in the robot’s end-effector.

### 2.2 Star 1: Learning and Applying Task-General Tool-Use Skills

In Star 1, our framework categorizes task demonstrations using our taxonomy and leverages taxonomic knowledge of the identified category to learn tool-use skills (i.e., the contact poses and trajectories) and generate actions with goals not seen in the training samples. In the following sections, we describe how tool-use skills are learned (Section 2.2.1) and applied to novel task configurations (Section 2.2.2). In Section 2.2.1, we detail the simulated demonstrations used to train the skills evaluated in this study. Subsequently, we discuss how demonstrations are categorized using our task taxonomy and how the corresponding taxonomic knowledge is leveraged to learn trajectory (Section 2.2.1.1) and contact pose (Section 2.2.1.2) representations. In Section 2.2.2, we detail how the system utilizes new task configurations to apply learned skills by generating new trajectories and contact poses.

#### 2.2.1 Learning Tool-Use Skills

The input data required by our algorithm include the start and goal poses of the manipulanda in the world frame and the tool trajectories as the keyframes in the world frame. Twenty simulated training samples per task were provided. Training samples were obtained with kinematic teaching of keyframe demonstrations in simulation. Each sample was a single demonstration of a task using a source tool and manipulandum. The samples were assumed to be successful demonstrations of a task, as no sophisticated outlier removal methods were utilized.

With the start and goal poses of the manipulanda, the system can infer the category of task being demonstrated to be used to guide the learning of trajectories and contact poses. If the goals of all demonstrations are zero vectors, then this task is a Non-Pose-Based Task. Otherwise, it is a Pose-Based Task. If it is the latter, the goals are converted to the manipulandum frame (i.e., the manipulanda frame is the start pose of the manipulanda) and are clustered based on the Euclidean distance between ω parts and the Euclidean distance between v parts of sample screw axes. If a unique cluster is found, then this task is considered a Finite-Goal Task. Otherwise, it is an Infinite-Goal Task.

##### 2.2.1.1 Learning Trajectories

The trajectory between two adjacent keyframes in a given demonstration is assumed to be interpolated, which may or may not be linear depending on rotational differences between the two frames. The keyframes can include only the start and goal poses of segments or any arbitrary number of midpoints. The keyframes are first merged into segments automatically. The different components of the trajectory are then identified by the framework. However, each component is assumed to have the same shape across demonstrations except for the functional component. Given a demonstrated trajectory comprising keyframes, the framework first groups the keyframes into segments with similar transformations between keyframes (i.e., the grouping stage). A component might be missing for different types of tasks, which is identified during this grouping stage. Subsequently, each segment, or partial segment, is then parametrized with the appropriate component and represented with $T_{start-prep}^{start-func}$ , $T_{end-prep}^{start-func}$ , {(screw axis S,  angles θ)} , and $T_{end-fin}^{end-func}$ ), as illustrated in Figure 2A (i.e., the parametrization stage).

The first step in the grouping stage is to identify the preparation component and the finishing component, which is to find the start pose $T_{start-prep}^{world}$ and the goal pose $T_{end-prep}^{world}$ (which is also the start of the contact component $T_{start-con}^{world}$ ) of the preparation component, and the start pose $T_{start-fin}^{world}$ (which is also the end of the functional component $T_{end-func}^{world}$ ) and the end pose $T_{end-fin}^{world}$ of the finishing component. To do this, the transformations between keyframes in the world frame are converted to the screw motion representation. Adjacent transformations with similar screw axes are merged. The similarity is evaluated with the Euclidean distance between ω parts and the Euclidean distance between v parts of sample screw axes. The merging is done by averaging the screw axis and summing the angles. After the merging, the first segment is assumed to be the preparation component, while the last is assumed to be the finishing component. The start and end poses of these components can thus be found.

The second step in the grouping stage is to identify the other components. For Non-Pose-Based Tasks, the rest of the segments are assumed to be the functional component, and the contact component of this type of task is assumed to be a segment with no transformations. For Pose-Based Tasks, the contact poses are assumed to be unchanged once the tool contacts the manipulanda. Therefore, the start of the functional component $T_{start-func}^{world}$ (which is also the end of the contact component $T_{end-con}^{world}$ ) can be obtained with $T_{start-man}^{world}\times {\left(T_{end-man}^{world}\right)}^{-1}\times T_{end-fin}^{world}$ . Since the start (i.e., the end of the preparation component) and the end (i.e., the start of the functional component) poses of the contact component are known, the contact component is found by interpolating these poses, which is obtained by calculating the screw axis of the transformation between the start and end poses and sampling angles with 1-degree intervals. Although the start and end poses of the functional component are known, the functional component is not a simple interpolation as it may need to follow a certain trajectory. Therefore, the algorithm allocates the remaining segments to the functional component, after excluding the partial segment belonging to the contact component. The partial segment is found by identifying the overlap between the first proceeding segment of the preparation component and the contact component.

In the parametrization stage, the keyframes are converted to different reference frames for easy application. The start and end poses of the preparation components are converted to the frame of the start pose of the functional component, resulting in $T_{start-prep}^{start-func}$ and $T_{end-prep}^{start-func}$ , respectively. The end pose of the finishing component is converted to the frame of the end pose of the functional component, which is $T_{end-fin}^{end-func}$ . If multiple segments comprise the functional component, each segment is represented with screw motion, and the start pose of this segment is used as the reference frame. As a result, the trajectory of a demonstration is represented using $T_{start-prep}^{start-func}$ , $T_{end-prep}^{start-func}$ , {(screw axis S,  angles θ)} , and $T_{end-fin}^{end-func}$ .

The next step of the parametrization stage is to find a template from all the training samples. The functional components of Infinite-Goal Tasks are ignored, as they are determined by the goal, rather than a shared trajectory template. For the rest of the tasks, the number of the segments comprising the functional component should be the same for each task. For the minority of demonstrations that are inconsistent with the number of segments that the majority of the demonstrations are associated with, those samples are excluded. For the remaining valid training samples, each segment of the component derived from different demonstrations is averaged. The transformations $T_{start-prep}^{start-func}$ , $T_{end-prep}^{start-func}$ , and $T_{end-fin}^{end-func}$ are also averaged from each demonstration.

##### 2.2.1.2 Learning Contact Poses

The current algorithm assumes a single contact area on the source tool when performing the same task, which could be relaxed in future studies. The contact area of the tool and the manipulandum were determined by proximity. For Infinite-Goal Tasks like object pushing where task success is contingent on the goals of the manipulandum, a change-of-basis of the start pose of the manipulandum is performed in order to incorporate the goal into its representation so that the contact poses are goal-based. The demonstrated contact poses are then converted to our representation of a class of contact poses using $S_{cp}^{tool}$ , $T_{man}^{tool}$ , and a group of {θ} .

For Infinite-Goal Tasks, we perform a change-of-basis on the start poses of the manipulandum before calculating the contact poses in order to account for the goal-directed nature of these tasks. The x axis is chosen to be the moving direction of the manipulanda, which is the normalized v part of a screw axis representing the transformation of the manipulandum from the start to the goal in the world frame. The z axis is chosen to be the direction of standard gravity. If the x axis and the z axis are parallel, an arbitrary direction is chosen ahead of time which is not parallel to the standard gravity. The y axis is obtained using the right-hand rule, which is the cross product x and z . To ensure the perpendicularity between x and z , the z axis is recalculated with the cross product of x and y . The position of the manipulanda remained the perceived position.

The contact pose of each demonstration $T_{man}^{tool}$ is obtained by ${\left(T_{start-func}^{world}\right)}^{-1}\times T_{start-man}^{world}$ where $T_{start-func}^{world}$ is the tool pose at the start of the functional component and $T_{start-man}^{world}$ is the start pose of the manipulanda. Then the contact poses from each demonstration are converted to our representation, a class of contact poses. The axis between any two contact poses is calculated, and the poses whose axis deviates too much from the majority of axes are excluded. An arbitrary pose, generally the pose of the first demonstration, is chosen as the origin $T_{man}^{tool}$ . The transformations between valid contact poses and this origin are calculated in the origin frame and represented using screw motion. The averaged axis $S_{cp}^{tool}$ is used as the axis of this class. For the angles obtained, if the Kolmogorov–Smirnov test on the group of angles showed no significant difference from a uniform distribution, then the range of this angle is used to represent {θ} . Otherwise, the groups of angles are clustered using density-based spatial clustering of applications with noise (DBSCAN; Ester et al., 1996), and the mean of each cluster is included in {θ} .

#### 2.2.2 Applying Tool-Use Skills

To apply the learned tool-use skills with the source tool and manipulanda, configurations of a task should be provided, which includes the start pose $T_{start-man}^{world}$ and the goal pose $T_{goal-man}^{world}$ of the manipulandum $T_{goal-man}^{world}$ . The goal pose $T_{goal-man}^{world}$ can be provided by perception (e.g., placed at the desired location) or by the experimenter in the form of a transformation matrix. The start pose $T_{start-man}^{world}$ is always perceived. The goal is always assumed valid for the given task and could be achieved by the given tool.

To use a tool, the contact poses and tool trajectories should be found. The contact poses are generated based on learned contact poses and taxonomic knowledge. Since multiple possible contact poses $T_{man}^{tool}$ exist for each task, multiple corresponding tool trajectories are generated. These tool trajectories are then converted into end-effector trajectories to be executed by the robot given the current perceived tool grasping pose. Trajectories are considered candidates if their functional components can be executed since the complete execution of the functional component is crucial to performing a task. The final trajectory is chosen from the candidates that minimize the required joint changes. If none of the functional components can be executed in full, the robot simply aborts execution. Otherwise, the robot attempts to execute as many components or partial components as possible since the full execution of other components is not central to successfully completing the task.

##### 2.2.2.1 Trajectory Generation

Given a contact pose $T_{man}^{tool}$ obtained from above, which is equivalent to $T_{start-man}^{start-func}$ , and the start pose $T_{start-man}^{world}$ and the goal pose $T_{goal-man}^{world}$ of a manipulandum, the start $T_{start-prep}^{start-man}$ and the end $T_{end-prep}^{start-man}$ of the preparation component in the manipulandum frame are calculated using the learned trajectories by ${\left(T_{start-man}^{start-func}\right)}^{-1}\times T_{start-prep}^{start-func}$ and ${\left(T_{start-man}^{start-func}\right)}^{-1}\times T_{end-prep}^{start-func}$ (the information from the learned trajectories is labeled with an enclosed rectangle), respectively. The preparation component in the manipulandum frame is then found by finding the interpolation between its start and end poses. The contact component in the manipulandum frame is obtained using the same method, with its start pose being the end pose of the preparation component and the end pose being the start of the functional component. In terms of the functional component, each segment of {[screw axis S, angle θ]} is found by interpolating the learned trajectory {[screw axis S, angle θ]} and converting those transformations to the manipulandum frame for the Non-Pose-Based Task. For Finite-Goal Tasks, the length of the trajectory, which is the angle in the screw motion representation, is adjusted according to the goal while the learned shape described by the screw axis remains the same. For Infinite-Goal Tasks (e.g., pushing), both the shape and length are determined by the goal with the end pose of the functional component being ${\left(T_{start-man}^{world}\right)}^{-1}\times T_{end-man}^{world}\times {\left(T_{start-man}^{start-func}\right)}^{-1}$ , and the trajectory is found by interpolating the start and end poses. The end pose of the finishing component is calculated using the learned trajectory as $T_{end-func}^{start-man}\times T_{end-fin}^{end-func}$ . In the end, each pose in the trajectory $T_{pose}^{start-man}$ is converted to the world frame with $T_{start-man}^{world}\times T_{pose}^{start-man}$ . In the writing task, when a different scale of the trajectory (e.g., write a larger or smaller “R”) is requested, the angle θ in {(screw axis S,  angles θ)} is scaled if the screw axis represents translational changes only, otherwise the ν part of the S in {(screw axis S,  angles θ)} is scaled. This works because the screw axis is in the previous pose’s frame, and the ν represents the velocity at the origin. To rotate the trajectory (e.g., to produce a tilted “R”), one can simply rotate $T_{start-func}^{world}$ . The corresponding start and end poses of other components need to be updated accordingly.

##### 2.2.2.2 Contact Pose Estimation

For the learned class of contact poses whose {θ} is composed of discrete values, the contact pose in the matrix form corresponding to each value is calculated. If the {θ} is a range, the contact poses are treated as discrete values by sampling angles from the range by 1-degree intervals. For Pose-Based Tasks, the contact poses are adjusted along the tool-moving direction so that a tool is guaranteed to touch the manipulandum (e.g., when pushing, an irregular object may require a slightly different relative position between the tool and the manipulandum).

### 2.3 Star 2: Task-General Object Substitution

Star 2 utilizes the tool-use skills learned by Star 1 and calculates the appropriate contact poses by finding the alignment between the source and substitute objects (Section 2.3.1), and adjusts the tool trajectory by leveraging the relevant taxonomic knowledge identified for each category of tasks (2.3.2). Star 2 requires the same manual inputs as the application in Star 1, which include the start and goal poses of the manipulanda, the desired number of circles for the stirring task, and the desired scale and rotation of the written letter for the writing task, as well as the grasping of the tool.

#### 2.3.1 Three-Step Alignment Algorithm

For all tasks except Infinite-Goal Tasks whose contact poses additionally depend on the goals, contact poses are obtained by calculating the alignment between the source and substitute objects. When the two tools are of the same type or share a generic form factor such as two different types of hammers, often considering the entire shape of both tools (i.e., their global features) produces the best results. In the case of tasks like pushing where no generic tool form-factor exists, utilizing features like the contact area (i.e., local features) of the source tool is necessary. Therefore, we designed a three-step alignment algorithm that produces mappings between source and substitute objects using both global (step one) and local features (step two) and selects the most appropriate one (step three). Since we consider local features, object meshes need to be segmented prior to applying this algorithm. The application of the three-step mapping algorithm differs slightly for tools and manipulanda.

In order to segment a mesh, we utilized a method similar to that used in a previous study (Abelha and Guerin, 2017), using the shape diameter function (CDF) with the CGAL library^5^. The number of clusters k ranged from 2 to 8 with step 1, and the smoothness parameter λ ranged from 0.1 to 0.7 with step 0.1. Since no direct relation exists between the number of clusters k and the results of the segmentation, the number of clusters with the greatest number of results was chosen as $k_{chosen}$ . Since, in most instances, the object with only one cluster is undesirable, $k_{chosen}$ was allowed to be one only if the number of results with one cluster was significantly more than the number of clusters with the second greatest number of results. The segmentation was randomly chosen from all the segmentations with $k_{chosen}$ clusters due to similarity.

**FIGURE 3:**
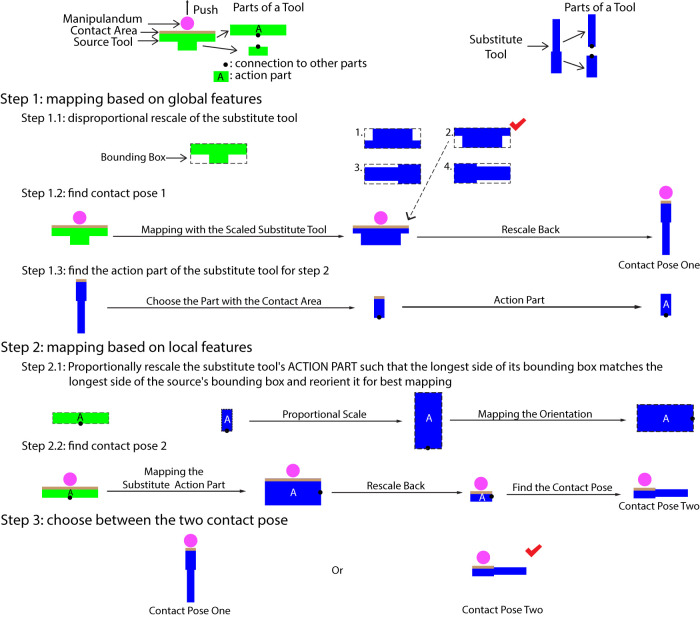
Mapping procedure for a hypothetical 2D tool substitution problem.

Figure 3 depicts and gives examples of our process for finding contact poses given segmented tool models, by finding the mapping $T_{sub-tool}^{src-tool}$ between the source and substitute meshes. When aligning the source and substitute objects in the first step, the substitute objects are rescaled disproportionally so that their bounding boxes share the same size as the bounding box of the source objects and reoriented along the axes of the bounding box. As an object can be rescaled and reoriented in multiple ways, the contact pose resulting from the rescaled and reoriented mesh that is most similar to the source object is chosen as contact pose one. The similarity is measured by the averaged minimum Euclidean distance between the points of the two point clouds when the centers of the two objects are aligned. The contact area on the substitute object is chosen by proximity to the contact area on the source object. The segment containing the contact area is chosen to be the action part which is used in step two. If the contact area is distributed across multiple segments, then the action part is chosen to be the contact area itself rather than any individual segment. As a result, we do not rely on the correctness of the segmentation. In the second step, in order to find contact pose two and the corresponding contact area, the two action parts are mapped in a similar manner, except that the substitute action part is rescaled proportionally, and the alignment of the two action parts uses modified iterative closest point (ICP) registration. In step three, of the two contact areas found in the two steps, the candidate with the highest similarity score is chosen along with its corresponding contact pose, and the mapping of the tools $T_{sub-tool}^{src-tool}$ is thus found.

The manipulanda do not need to be decomposed into action and grasping parts like tools do. Therefore, the contact area is used as the action part, and the algorithm to find the mapping poses of the substitute manipulanda $T_{sub-man}^{src-man}$ is otherwise the same as finding the mapping of the substitute tool. For Infinite-Goal Tasks, the mapping of the manipulanda is not needed since the geometric features of the manipulanda do not decide the mapping. Therefore, it is handled in the same manner as the source manipulanda in that the start pose is updated to incorporate the goal. The mapping, in this case, is set to be the identity matrix.

#### 2.3.2 Generating Tool Trajectories

Given the mapping resulting from the three-step alignment algorithm, the trajectory of the substitute tool can be found given the learned source tool trajectory with adjustments based on the taxonomic knowledge if necessary (see Section 2.2.2). With the obtained tool trajectory, the end-effector trajectory is calculated from the tool trajectory in the same way as Star 1, except that the functional component is rescaled based on the size of the substitute manipulandum relative to the source one for Non-Pose-Based tasks.

To find a candidate tool trajectory, an equivalent trajectory of the source tool acting upon an equivalent source manipulanda (i.e., the equivalent start pose and goal pose of the manipulandum is calculated with $T_{start-sub-man}^{world}\times {\left(T_{sub-man}^{src-man}\right)}^{-1}$ and $T_{end-sub-man}^{world}\times {\left(T_{sub-man}^{src-man}\right)}^{-1}$ , respectively) is first found. Then each pose of such a trajectory $T_{src-tool}^{src-man}$ is updated with ${\left(T_{sub-man}^{src-man}\right)}^{-1}\times T_{src-tool}^{source-man}\times T_{sub-tool}^{src-tool}$ which calculates the trajectory of the substitute tool in the substitute manipulandum frame. The trajectory is then converted to the world frame. For Non-Pose-Based Tasks, the functional component of the trajectory is rescaled based on the relative size of the longest dimension of the source and substitute manipulandum. Multiple candidate tool trajectories are found, each corresponding to a contact pose chosen in the same way as in Star 1. The final tool trajectory is chosen from the candidate tool trajectories in the same way as in Star 1.

### 2.4 Star 3: Tool-Use Transfer to Other Robot Platforms

As tool-use skills learned by Star 1 are represented independent of robot configurations, no additional algorithms were needed in order to enable skill transfer to different platforms that could perform the given task. This was assisted *via* the development of a perception system that obtains the 3D poses of the tools and manipulanda from RGB-D cameras, though in principle, any method that can accurately perceive these poses can be used. With the learned tool-use skills and the perceived grasping, we calculate the end-effector trajectories and control the robot by leveraging existing inverse kinematics and motion planning libraries. In order to simplify motion control across different robot platforms, we implemented a robot operating system node that uses the same interface to control all three robots. This interface can be easily extended to accommodate more platforms.

The same mechanisms of partially executing a trajectory or completely aborting it mentioned in the Applying subsection of Star 1 also apply when the platforms being transferred to cannot execute the generated actions. Moreover, learning a class of contact poses also helps with finding viable solutions on different platforms. For example, in the knocking task, the robot can choose to approach a manipulandum from any orientation, even those that did not appear in the training set, which increases the viable kinematic solutions when a robot searches for motion planning.

## 3 Results

TRI-STAR uses raw sensor data for perception and demonstrated Star 1 with seven tasks trained with minimal training samples *via* learning from demonstration (Argall et al., 2009). We tested Star 2 by providing three substitute tools and three manipulanda for each task. Finally, we conducted experiments for Star 3 that transferred the learned skills to two other robot platforms with different kinematic configurations. The raw data can be found in Supplementary Table S1.

### 3.1 Star 1: Learning and Applying Task-General Tool-Use Skills

**FIGURE 4:**
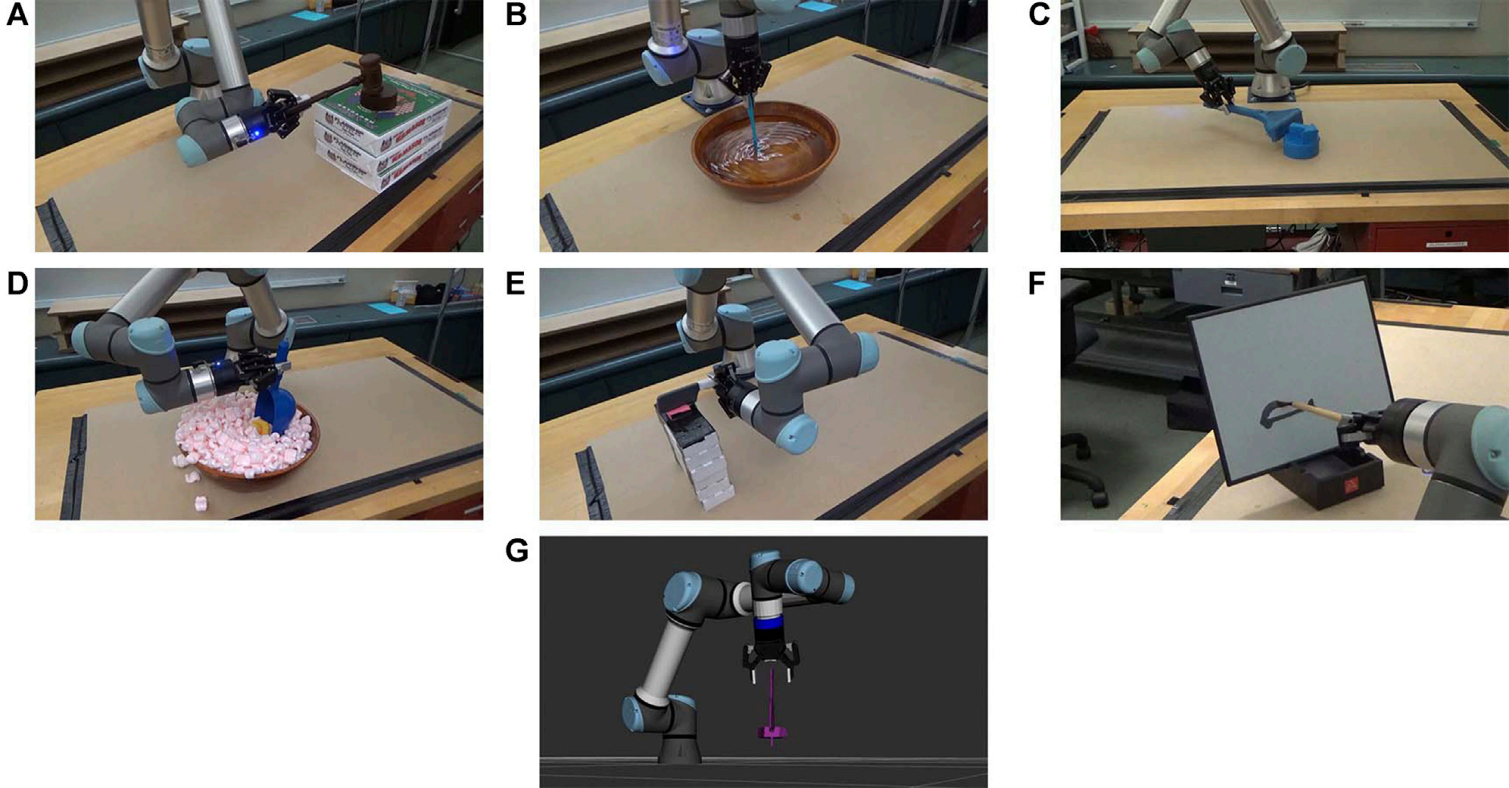
Demonstration of the variety of tasks learned by the robots using source objects. Star 1 tested a robot learning a wide range of tasks, including **(A)** knocking, **(B)** stirring, **(C)** pushing, **(D)** scooping, **(E)** cutting, **(F)** writing, and **(G)** screw-driving.

**FIGURE 5:**
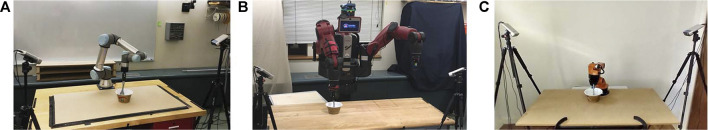
Workspaces. The workspace of **(A)** UR5e, **(B)** Baxter, and **(C)** the Kuka youBot robot are similar. Two Azure Kinect RGB-D sensors are placed on the sides of the workspace.

Figures 4A–G shows an example from each of the seven tasks with the source tools and manipulanda, and Figure 5 shows the testing environment. Six of the seven tasks were tested on a UR5e robot, and the screw-driving task was demonstrated on a simulated UR5e due to the higher perception accuracy required to align the tip of a screwdriver to the slot on the head of a screw. All tasks tested on the physical robot were evaluated quantitatively except for the writing task, which was included for demonstration purposes only. Creating quantitative metrics was sometimes challenging; while the pushing task could be evaluated with translation errors to the goal as had been done previously (Fitzgerald et al., 2019; Xie et al., 2019), other tasks were previously reported with only binary success/failure results (Pastor et al., 2009; Brandi et al., 2014) or success rates over multiple trials (Gajewski et al., 2019; Fang et al., 2020). When evaluating performance quantitatively, we used stricter methods (e.g., using loudness in decibels for the knocking task) when possible.

**FIGURE 6:**
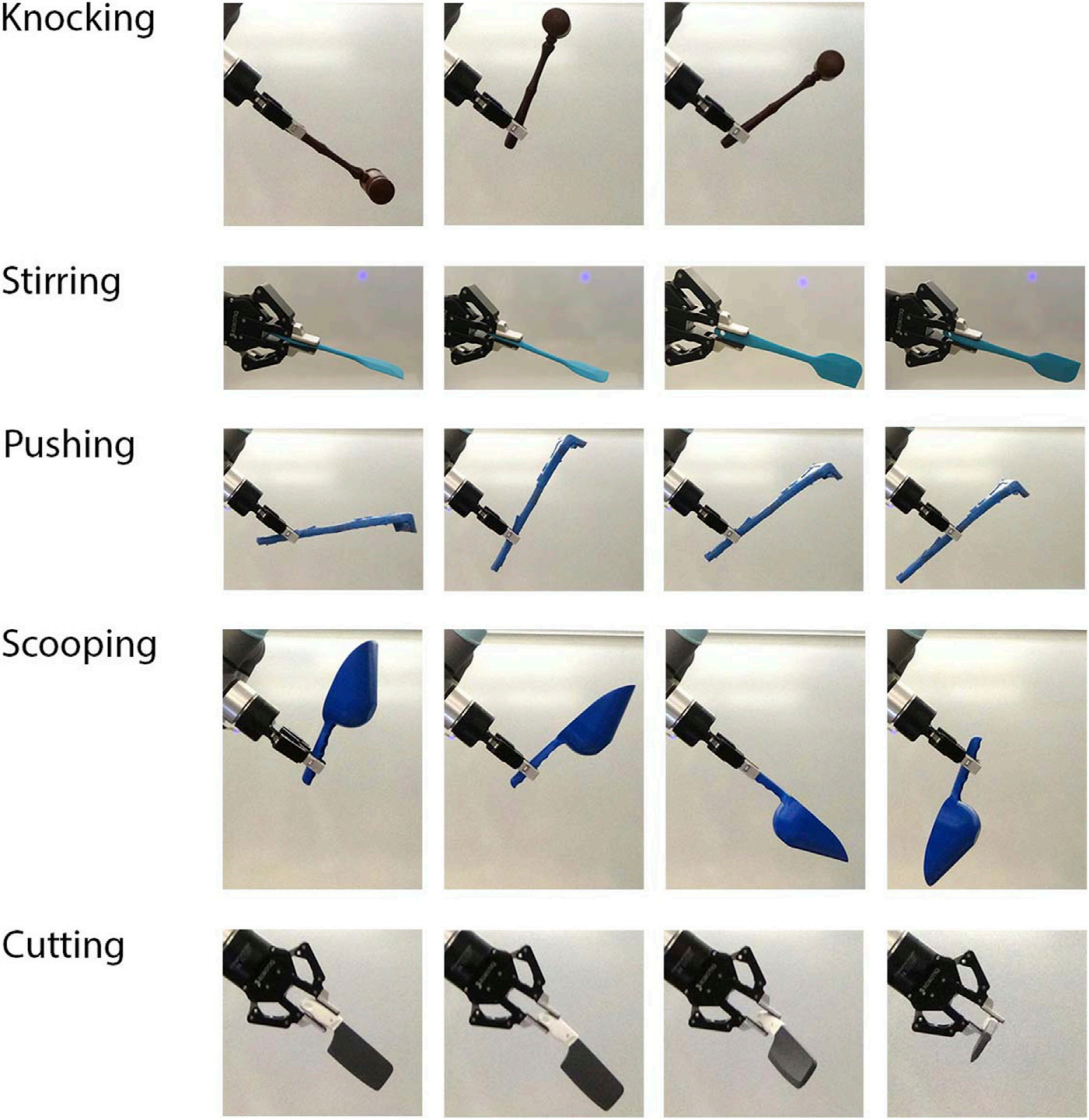
Different grasping poses of the source tools (Star 1). For each task, that is, knocking, stirring, pushing, scooping, and cutting, at least three different grasping poses were tested.

**FIGURE 7:**
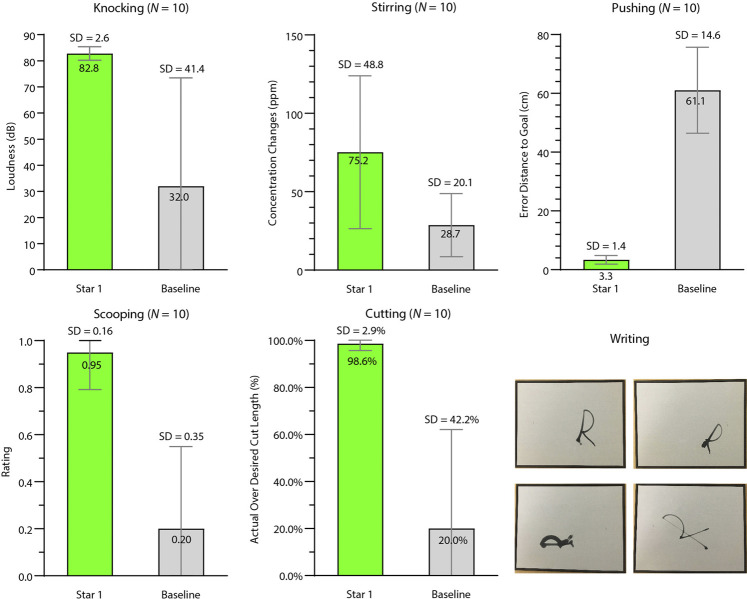
Results of learning source tools with source manipulanda (Star 1). We compared Star 1 (green) performance against a baseline (gray) for knocking, stirring, pushing, scooping, and cutting. The pictures at the bottom right show the demonstrations of the writing task. The top left is an “R” using the same scale and rotation as the training sample. The top right, bottom left, and bottom right “R”s used the following scales and orientations: scale = 1.0, orientation = 270 ° ; scale = 0.8, orientation = 30 ° ; scale = 1.5, orientation = 300 ° .

The five tasks analyzed quantitatively were also compared with a baseline condition. We designed the baseline condition in accordance with the common practice across task-general tool-use learning frameworks of using the gripper pose as a proxy for the tool pose. Therefore, in the baseline condition, the robot repeated an end-effector trajectory in the task space of a training sample chosen randomly. For the five tasks, we tested ten trials per task per condition. Trials in which the robot was not able to follow the commanded trajectories were excluded. The start and goal poses of the manipulanda were altered in each trial. In both the experimental and baseline conditions, the robot held tools with various poses as shown in Figure 6, a complexity that was not present in other studies. These poses were provided to the robot by the experimenters in order to impose pose variety (see Section 1.4 for motivation), though in principle, TRI-STAR can accommodate autonomous grasping. Figure 7 summarizes the results, and Supplementary Video S1 shows demonstrations of the robot performing the learned tasks. Details of testing each task are described below.

#### 3.1.1 Knocking

The robot successfully completed the task in 10 out of 10 trials in the testing condition, while its performance in the baseline condition was 4 out of 10 trials. We also measured the sound of each knock on the manipulandum using the Sound Meter app with a Samsung tablet placed close to the manipulandum. The average decibels, including the reading from unsuccessful trials, of the testing condition [mean (M) = 82.79 decibel (dB), Standard Deviation (SD) = 2.58 dB] were higher than those of the baseline condition (M = 32.00 dB, SD = 41.44 dB).

#### 3.1.2 Stirring

0.25 tsp salt per liter was added to the room-temperature water and given several seconds to settle. The robot was allowed to stir for 1 min or five circles, whichever lasted longer. Due to kinematic constraints, the grasps in the testing conditions were similar to the training pose. This constraint, along with the enforced grasping pose consistency across training and baseline conditions, resulted in both training and testing conditions completing 10 of 10 trials. We also measured the concentration changes in part per million (ppm) before and after the stirring using a total dissolved solids meter. More salt dissolved in the testing condition (M = 75.20 ppm, SD = 48.79 ppm) than in the baseline condition (M = 28.70 ppm, SD = 20.10 ppm).

#### 3.1.3 Pushing

The manipulandum was pushed closer to the goal position in the testing condition (translation error: M = 3.36 centimeters (cm), SD = 1.45 cm) than in the baseline condition (M = 61.06 cm, SD = 14.62 cm). Our translation error in the testing condition is consistent with a recent study (Xie et al., 2019; M = 6.37 cm, SD = 5.33 cm) which also utilized perceptual data from raw sensor readings. The translation errors were mainly due to perception errors. This is supported by the significantly reduced translation error (M = 0.013 cm, SD = 0.0074 cm) observed when performing the same experiments using a simulated UR5e robot with perfect perception.

#### 3.1.4 Scooping

The performance was rated as 1 if the robot successfully scooped the manipulandum, 0.5 if the rubber duck slipped away but the robot scooped surrounding packing material, and 0 if the robot failed to scoop anything. The robot scooped the manipulandum more successfully in the testing condition (M = 0.95, SD = 0.16) than in the baseline condition (M = 0.20, SD = 0.35).

#### 3.1.5 Cutting

We measured the percentage length of the actual cut over the length of the intended cut. Even with a relaxed criterion accepting cuts as shallow as 1 mm in the baseline condition, the robot cut the putty more thoroughly in the testing condition (M = 98.62%, SD = 2.91%) than in the baseline condition (M = 20.00%, SD = 42.16%).

#### 3.1.6 Writing

The robot was required to write the trained letter “R” and the letter “R” with untrained scales and orientations. Figure 7 shows various letters “R” that the robot wrote.

#### 3.1.7 Screw-Driving

The robot in simulation completed the task successfully.

### 3.2 Star 2: Task-General Object Substitution

**FIGURE 8:**
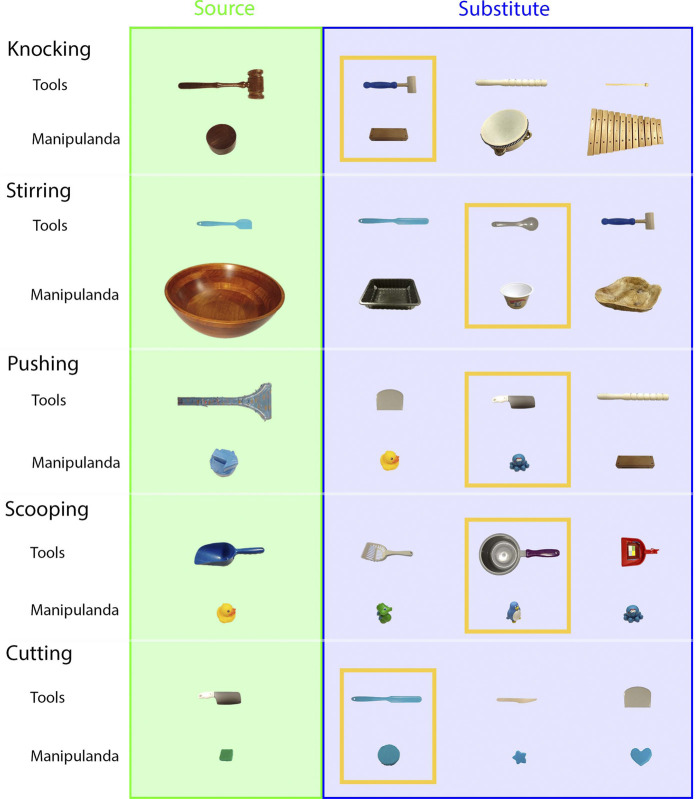
Substitute objects (Star 2). For each task, that is, knocking, stirring, pushing, scooping, and cutting, three substitute tools and three substitute manipulanda were included in testing. The objects in the yellow frames were used as source objects in Star 3.

**FIGURE 9:**
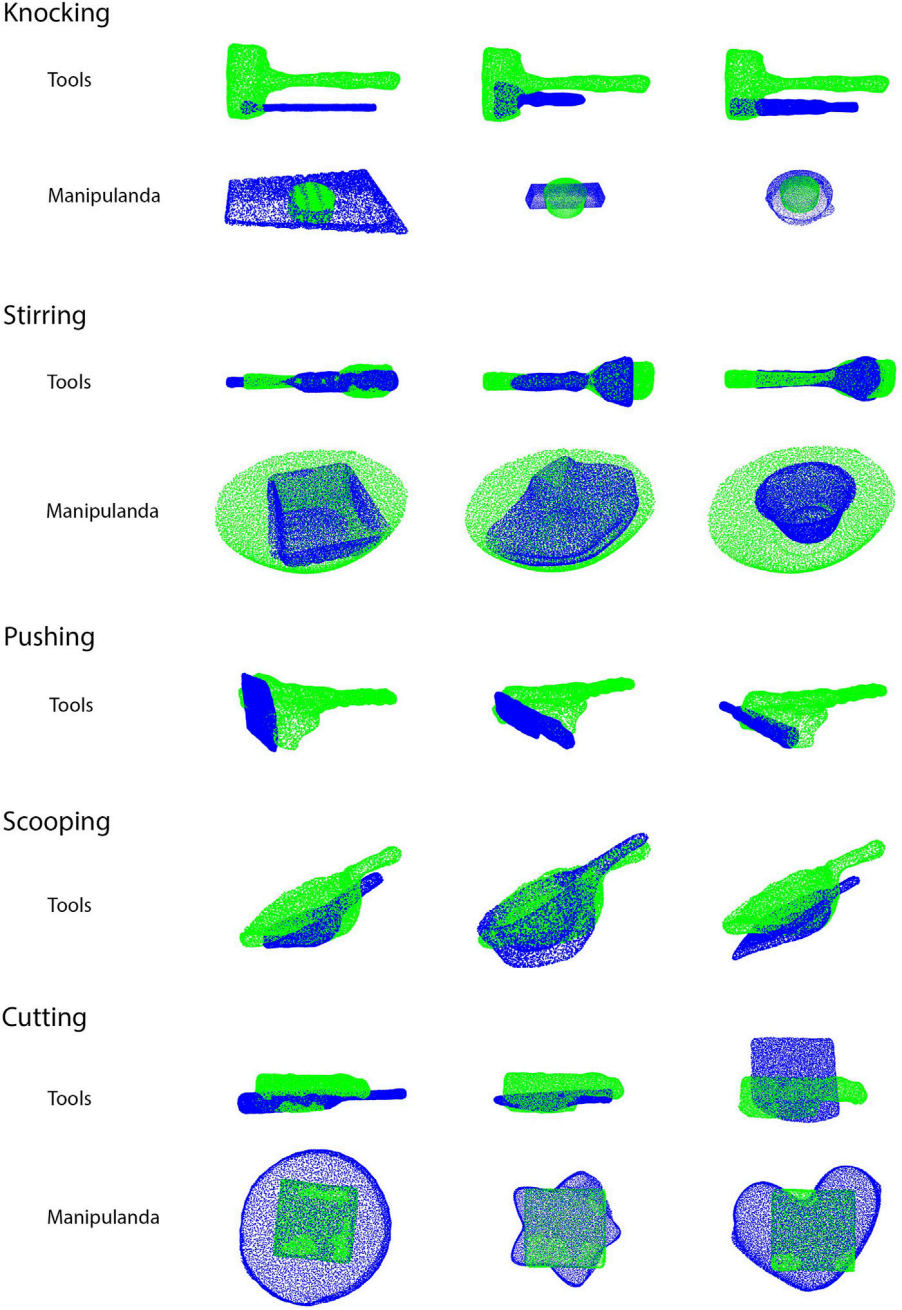
Results of mapping substitute objects to source objects (Star 2). The green point clouds are the source objects while the blue point clouds are the substitute objects. Manipulandum substitution for the pushing and scooping task is not geometry-dependent, but goal-dependent, and therefore, the mapping results are excluded in the figure.

**FIGURE 10:**
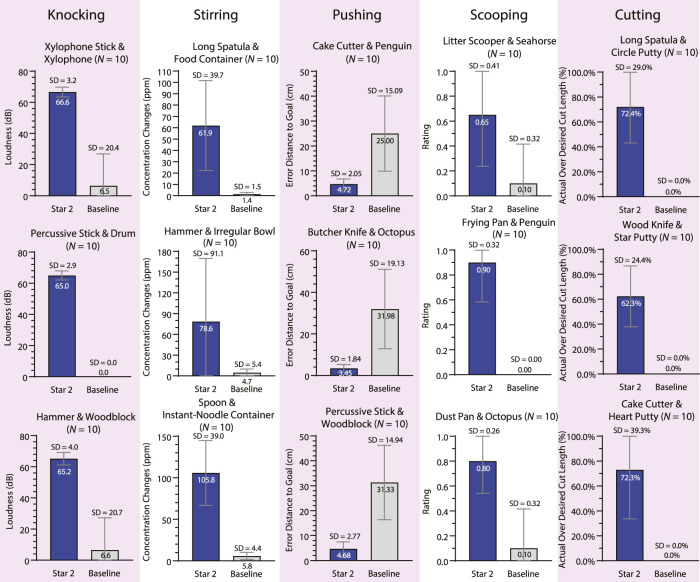
Results of tool substitution and manipulandum substitution (Star 2). The bar graphs show the results of using the substitute objects to perform knocking, stirring, pushing, scooping, and cutting. The bars compare Star 2’s (blue) performance against the baseline (gray).

Five tasks (knocking, stirring, pushing, scooping, and cutting) were tested on a UR5e robot. Other than using substitute objects, the experiments and evaluation in Star 2 were the same as those performed in Star 1. For each task, three pairs of substitute objects were tested, and all objects were appropriate for the tasks. In the baseline condition, a random contact area and a contact pose were chosen on each of the substitute objects. The trajectories were generated using the same method as the testing condition. Figure 8 shows the source and substitute objects. Figure 9 shows the mapping result of each substitute object with the source object in each task. Figure 10 summarizes the results of the five tasks. Supplementary Video S2 shows the robot performing tasks with substitute tools and manipulanda. Details of each task are described below.

#### 3.2.1 Knocking

All three substitute tools successfully struck the substitute manipulanda in all trials in the testing condition, while the performance dropped significantly in the baseline condition (i.e., at most 1 out of 10 trials for each tool–manipulandum combination). In a previous study with a similar task (Fang et al., 2020), the highest success rate on nail-hammering was 86.7% of all the substitute tools with tens of thousands of training samples. In the testing condition, the average loudness in the testing condition (M = 65.62 dB, SD = 3.35 dB) was higher than that of the baseline condition (M = 4.34 dB, SD = 16.50 dB), while the loudness was not measured in the previous study.

#### 3.2.2 Stirring

All three substitute tools successfully stirred the room-temperature salted water in the substitute containers in all trials in the testing condition, while all substitute tools failed to stir in the baseline condition. More salt dissolved in the testing condition (concentration change: M = 82.10 ppm, SD = 62.29 ppm) than in the baseline condition (M = 3.97 ppm, SD = 4.43 ppm). We did not encounter another study that performed a similar task.

#### 3.2.3 Pushing

The manipulanda were pushed closer to the goal in the testing condition (translation error: M = 4.28 cm, SD = 2.26 cm) than in the baseline condition (M = 29.44 cm, SD = 16.24 cm). In a previous study that also used raw sensor data to perceive the environment (Xie et al., 2019), the translation error using substitute tools and source manipulanda was similar (M = 5.56 cm, SD = 4.13 cm) to that in the current study but required more than 104 training samples.

#### 3.2.4 Scooping

The substitute tools scooped the substitute manipulanda more successfully in the testing condition (rating: M = 0.78, SD = 0.34) than in the baseline condition (M = 0.07, SD = 0.25). In a previous study (Gajewski et al., 2019), the scooping task was tested only in simulation with substitute tools and source manipulanda, and no quantitative results (i.e., success rate) were provided.

#### 3.2.5 Cutting

The robot cut the manipulanda more thoroughly in the testing condition (cut length percentage: M = 78.33%, SD = 33.95%) than in the baseline condition (M = 6.67%, SD = 25.37%) even with relaxed criteria in the baseline condition as mentioned in the Star 1 evaluation. In a previous study (Gajewski et al., 2019), the cutting task was tested only in simulation with substitute tools and source manipulanda, and no quantitative results (e.g., success rate) were provided.

### 3.3 Star 3: Tool-Use Transfer to Other Robot Platforms

**FIGURE 11:**
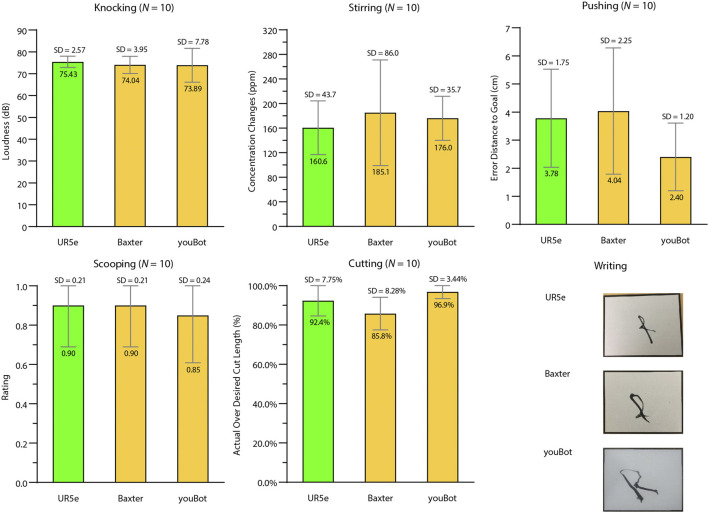
Results of tool-use skill generalization across robot platforms (Star 3). The bar graphs include results of the UR5e (green), Baxter (yellow), and youBot (yellow) using the source tool/manipulandum combinations for knocking, stirring, pushing, scooping, and cutting. The pictures at the bottom right demonstrate different robots writing “R” with trained scale and orientation.

Six tasks (pushing, stirring, knocking, cutting, scooping, and writing) were used to test skill transfer from a UR5e robot to both a Baxter robot and a Kuka youBot without additional training. Due to the size and payload limitations of Baxter and youBot, source tools different from Star 1 were chosen. The experiments were similar to the ones in Star 1. However, no baseline conditions were included in Star 3, and no comparisons were made with other studies since we did not encounter similar studies. Figures 5B,C show the testing environment of Baxter and youBot. The objects in the yellow frames of Figure 8 are the objects tested in Star 3. Star 3 only considered scenarios that the new platforms could complete if they were trained in the same way as the source platform. Therefore, the task configurations of all experiments were within the feasible workspace of the new robots. Figure 11 summarizes the results. Supplementary Video S3 shows both robots performing different tasks.

#### 3.3.1 Knocking

All three robots successfully completed all trials. The loudness created by the UR5e (M = 75.43 dB, SD = 2.57 dB), Baxter (M = 74.04 dB, SD = 3.95 dB), and youBot (M = 73.89 dB, SD = 7.78 dB) were similar.

#### 3.3.2 Stirring

All three robots successfully completed all trials. The concentration changes of the stirs by Baxter (M = 185.10 ppm, SD = 86.01 ppm) and youBot (M = 176.00 ppm, SD = 35.74 ppm) were slightly higher than the stirs by the UR5e (M = 160.60 ppm, SD = 43.71 ppm).

#### 3.3.3 Pushing

YouBot (translation error: M = 2.40 cm, SD = 1.02 cm) pushed the manipulanda slightly closer to the goal than UR5e (M = 3.78 cm, SD = 1.74 cm) and Baxter (M = 4.04 cm, SD = 2.25 cm), which was because of the shorter pushing length by youBot due to limited maximum reach compared with UR5e and Baxter.

#### 3.3.4 Scooping

UR5e (ratings: M = 0.90, SD = 0.21), Baxter (M = 0.90, SD = 0.21), and youBot (M = 0.85, SD = 0.24) performed equally well.

#### 3.3.5 Cutting

The average cut length percentage cut of UR5e (M = 92.39%, SD = 7.75%) and youBot (M = 96.92%, SD = 3.44%) was slightly longer than that of Baxter (M = 85.83%, SD = 8.28%), which was due to the difficulty in securing the spatula tightly in Baxter’s gripper.

#### 3.3.6 Writing

All three robots were able to repeat the letter “R.” Figure 11 shows the letter “R” with the trained scale and orientation written by the three robots.

#### 3.3.7 Screw-Driving

All three robots in simulation completed the task successfully.

## 4 Discussion

The results showed that the TRI-STAR framework learned a wide range of tasks, generalized the learned skills to substitute tools and manipulanda, and transferred the learned skills across robot platforms. This was achieved by using our task-oriented approach, which includes a goal-based task taxonomy and identified taxonomic knowledge which specifies knowledge shared across tasks that belong to particular task categories and minimizes the need for knowledge to be defined on a per task basis. We center our discussion around the ways our framework improves upon the state of the art in task-general tool use but also identify limitations of our approach, as summarized in Table 1.

**TABLE 1:** Comparing different tool-use frameworks: transfer by correction (Fitzgerald et al., 2019), kPAM and kPAM 2.0 (Manuelli et al., 2019; Gao and Tedrake, 2021), warping (Brandi et al., 2014), TOG-net (Fang et al., 2020), p-tools (Abelha and Guerin, 2017; Gajewski et al., 2019), GVF (Xie et al., 2019), and TRI-STAR (ours). The different frameworks are listed by row, ordered by relevancy to our work. Cell shading indicates how desirable a demonstrated feature of the corresponding framework is, with darker shading indicating higher desirability. Comparison of Task-Generality and Data Efficiency. The tables show that not all frameworks are task-general (e.g., do not require pre-specified knowledge for each individual task). Moreover, TRI-STAR was demonstrated with a wider range of tasks than other frameworks. Additionally, TRI-STAR requires fewer training samples per task than other task-general frameworks.

	Task-general?	Tool-use tasks tested		# of training samples
		Physical robot	Simulation	
Transfer by correction	N/A^5^	Hooking	-	1 trajectory per source tool and 2 poses per substitute tool for within-task transfer
		Sweeping		
		Hammering		
kPAM	No	Whiteboard-wiping	-	10–30 per task
		Peg-hold insertion		
Warping	No	Pouring	Pouring	10–50 per task
			Filling	
TOG-net	Yes	Hammering	Hammering	18,000 in total
		Sweeping	Sweeping	
p-tools	Yes	Scraping	Cutting	5,000 in total
			Scooping	
			Scraping	
			Hammering	
			Rolling dough	
			Lifting	
GVF	Yes	Sweeping	-	24,006 in total
		Scraping		
		Wiping		
TRI-STAR (ours)	Yes	Pushing	Driving-screws	20 per task
		Scooping		
		Cutting		
		Writing		
		Stirring		
		Knocking		

### 4.1 Contribution 1: Task-Generality

TRI-STAR is a task-general tool-use framework shown to learn, generalize, and transfer tool-use skills for a variety of everyday tasks. As shown in Table 1, not all tool-use algorithms are intended to be task-general as they assume pre-defined knowledge specific to individual tasks at either the learning or the generalization stage. Three advances made it possible for TRI-STAR to be task-general. First, we summarized taxonomic knowledge of tasks which enables a multitude of tasks to be learned efficiently, including potentially any undemonstrated tasks covered by one of the known taxonomic categories. Second, TRI-STAR can handle tasks with different contact pose requirements (e.g., pushing, knocking, and screw-driving) and different types of trajectories (e.g., circular periodic trajectories including stirring, linear trajectories including cutting, nonlinear trajectories including scooping, trajectories that could be either linear or nonlinear including pushing, and complex trajectories with both linear and nonlinear segments including writing), which allows a robot to work with a wide range of tasks. Third, TRI-STAR can generalize the tool-use skills to tasks whose substitute tools and manipulanda may be geometrically similar or geometrically distinct objects since we made no assumptions about the shape of the objects, unlike previous approaches (Brandi et al., 2014; Fang et al., 2020). An added benefit of generalizing tool-use skills to geometrically distinct objects is that it can allow a robot to improvise the use of objects such that an object not designed for a task could be used when desired objects are unavailable.

### 4.2 Contribution 2: Data Efficient

As shown in Table 1, the task-general framework typically required a large sample size. However, training with a large sample size is time-consuming and thus impractical in time-sensitive domains like search-and-rescue.^6^ By leveraging taxonomic knowledge identified for each task category, TRI-STAR required only 20 examples to learn each task, and no additional training samples were needed by Star 2 to generalize the usage to substitute objects or by Star 3 to transfer the skills to other platforms. In contrast, previous studies required over 5,000 (Gajewski et al., 2019), 18,000 (Fang et al., 2020), and 20,000 (Xie et al., 2019) training samples. The small set of training samples needed for each task makes it time-efficient for TRI-STAR to learn new tasks, and thus, it is easy to be deployed as an application in the real world. Moreover, TRI-STAR experienced only a minor loss in performance while significantly reducing the necessary training samples.

### 4.3 Contribution 3: Integrative Framework

We demonstrate TRI-STAR’s ability to handle all three stars, including tool-use learning, tool substitution, and tool-use transference to other platforms, as shown in Table 2. Previous studies on task-general tool-use focused on either tool-use learning or tool substitution and typically limited the types of objects considered (e.g., they only consider objects that share similar form-factors or only consider tool but not manipulanda substitution). Other tool-use studies tend to be customized to particular tasks, which makes adapting them for the wide variety of tasks a robot might realistically encounter challenging without significant modifications. In contrast, TRI-STAR not only enables all these functionalities within one integrative framework but also removes these limitations. Moreover, our framework encompasses an entire tool use–centric pipeline which includes important aspects often ignored in other studies such as tool–manipulandum contact pose learning and a perception system customized to the needs of tool use. Our framework covered important aspects that were not mentioned in previous studies, such as tool–manipulandum contact pose learning. We integrated all of these into TRI-STAR and showed its effectiveness with a wide range of tasks. Being an integrative framework makes it plausible for TRI-STAR to be deployed into real-world contexts.

**TABLE 2:** Comparing functionality. The table showed that TRI-STAR is an integrative framework with the most demonstrated functionality.

	Integrative framework		
	Star 1: learns tool use from demonstrations only	Star 2: able to utilize arbitrary tools/manipulanda	Star 3: demonstrated transfer without retraining to other robots
Transfer by correction	Yes	Yes, but requires human corrections for novel tools	No
kPAM	No	Yes, but limited to tools that share common form factors	No
Warping	Yes	Yes, but limited to tools/manipulandum with common form-factors	No
TOG-net	No	Yes, but limited to tools	No
p-tools	No	Yes, but limited to tools	No
GVF	Yes	Yes	No
TRI-STAR (ours)	Yes	Yes	Yes

Star 3 does not require additional algorithmic infrastructure to implement, but rather, updating a common representational schema (Cartesian trajectory) that is utilized in many tool-use studies. To automate research work, robots have been deployed in chemistry laboratories (Burger et al., 2020), where tasks and tools are standardized. The ability to transfer skills between robots could save researchers in each laboratory hundreds of hours of training time as skills could be shared across research laboratories. For robots in the factory or warehouse, it will be cost-efficient for skills to be transferred to new models without having to shut down the factory in order to debug compatibility-related problems. For other applications, platform-agnostic skill transfer would not merely be a convenience but could open entirely new applications. For example, for in-home robots, the prospect of training every single task by each individual is a nonstarter for most consumers, whereas having access to a shared library of skills may be more acceptable.

### 4.4 Limitations

While our results demonstrate the potential of our framework, it has limitations. Table 3 summarizes the major limitations. First, the robot used position control only, rather than force control or feedback control, to learn and complete tasks, which limits its effectiveness on tasks that require consideration of the forces being applied to the manipulanda such as nail-hammering, or tactile feedback such as inserting a key into a lock. Second, our framework only considers the geometric features of the tools and manipulanda and does not consider other properties (e.g., material, weight, and texture), which may hinder the robot’s ability to choose the most appropriate contact areas for tools like sandpaper that have a single abrasive surface but are otherwise geometrically uniform. Third, although our system calculated the grasping location on the tool, automatic grasping was not demonstrated in the evaluation.

**TABLE 3:** Comparison of major limitations. The table shows that while all frameworks considered only geometric properties of objects, some frameworks employed more sophisticated control techniques than TRI-STAR.

	Limitations		
	Velocity/torque control	Object features considered	Generate viable grasp poses?
Transfer by correction	No	Geometric only	No
kPAM	Yes		Yes
Warping	Yes		Unknown
TOG-net	No		Yes
p-tools	No		Yes, but not demonstrated during evaluation
GVF	No		Yes
TRI-STAR (ours)	No		Yes, but not demonstrated during evaluation

Other limitations also exist for TRI-STAR. First, our framework assumes that all objects, including relevant objects in the environment, are rigid bodies with no joints (i.e., have 0 DoF). This assumption does not allow a robot to handle common tools or manipulanda such as scissors or washcloths or to perform tool-use tasks on top of soft surfaces. Second, our framework relies on accurate visual perception and structured environments, which is a common problem for non–marker-based perception systems and is an impediment to handling tasks that require highly accurate perception, such as surgery. Third, object mapping relies on full 3D models though ideally, this system should perform mappings using only partial point cloud data of both geometrically similar and geometrically distinct objects. Fourth, TRI-STAR cannot learn the cause-and-effect relations (e.g., Brawer et al., 2020) that comprise taxonomic knowledge, which does not allow it to, for example, automatically choose between the actions required to stir a liquid versus a heavier mixture like a batter.

## Data Availability

The original contributions presented in the study are included in the article/Supplementary Material; further inquiries can be directed to the corresponding authors.

## Footnotes

1 Source code located at https://github.com/ScazLab/Frontiers_Robot_Tool_Use.git.

2
https://www.autodesk.com/.

3
https://github.com/3DLIRIOUS/MeshLabXML.

4
https://www.meshlab.net/.

5
https://doc.cgal.org/latest/Surface_mesh_segmentation/index.html.

6 Although within-task transfer to novel tools requires additional data, across-task transfer does not.
